# Supplementary material for: Microscopic polyangiitis plasma-derived exosomal miR-1287-5p induces endothelial inflammatory injury and neutrophil adhesion by targeting CBL
Source: PeerJ. 2023 Jan 27;11:e14579. doi: 10.7717/peerj.14579 (PMC9885867; doi:10.7717/peerj.14579)

Picture 1

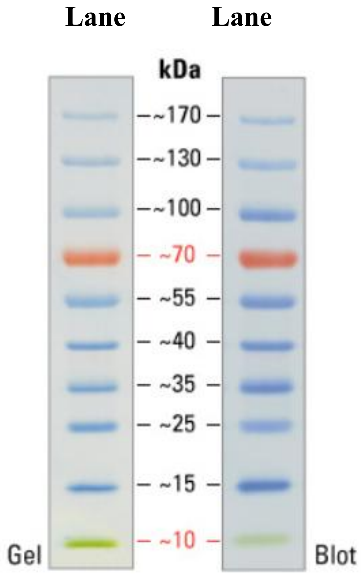

Picture 2

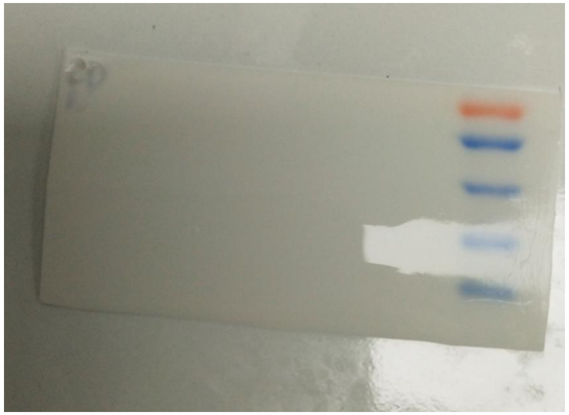

Picture 3

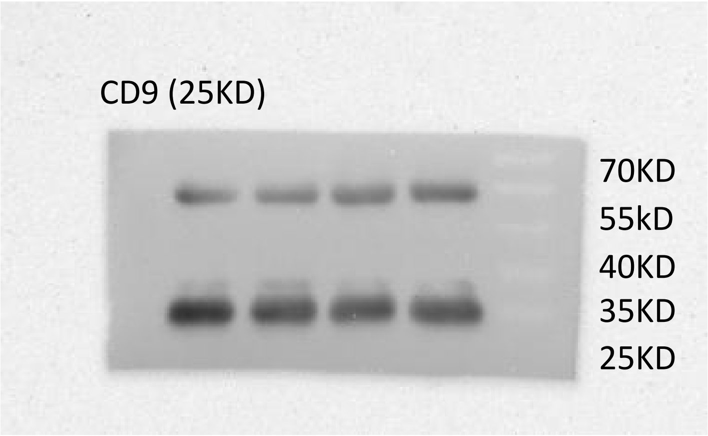

Lane 1 is the schematic diagram of the marker we selected (Thermo), and we found that the marker at the 70kD position is red. Picture 2 is a PVDF membrane incubated with CD9, picture 3 is the picture of the western blot. The top red brand in picture 2 is 70kD, and we found the molecular weight of CD9 is 25kD.

CBL (120KD)

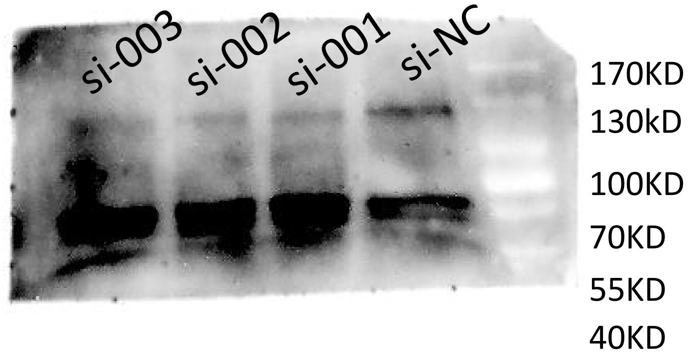

β-tublin (55KD)

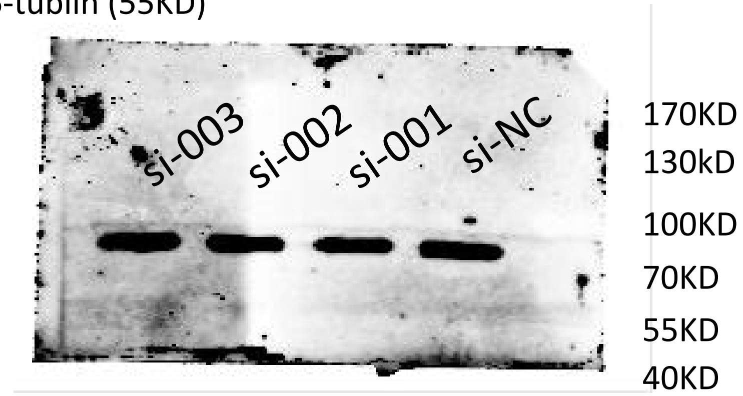

Exosome marker protein

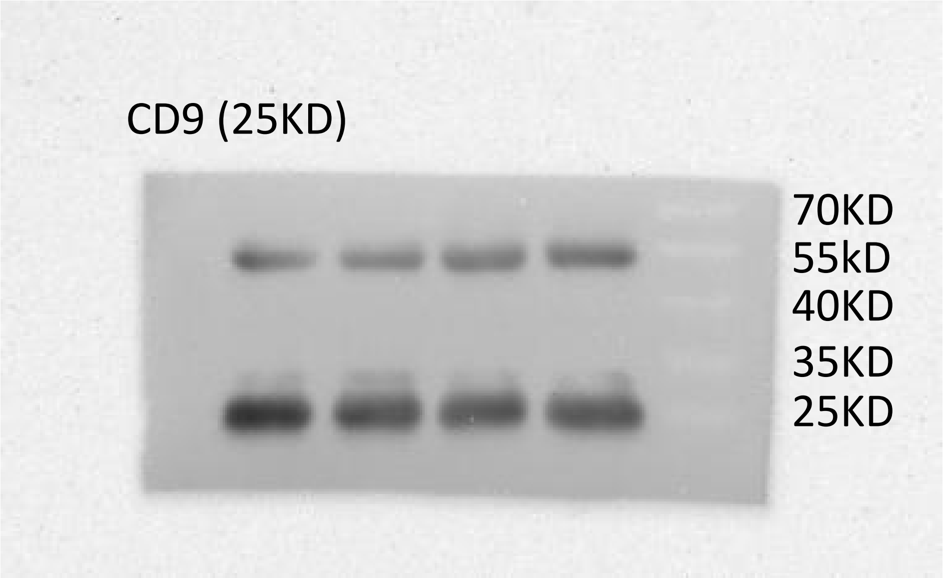

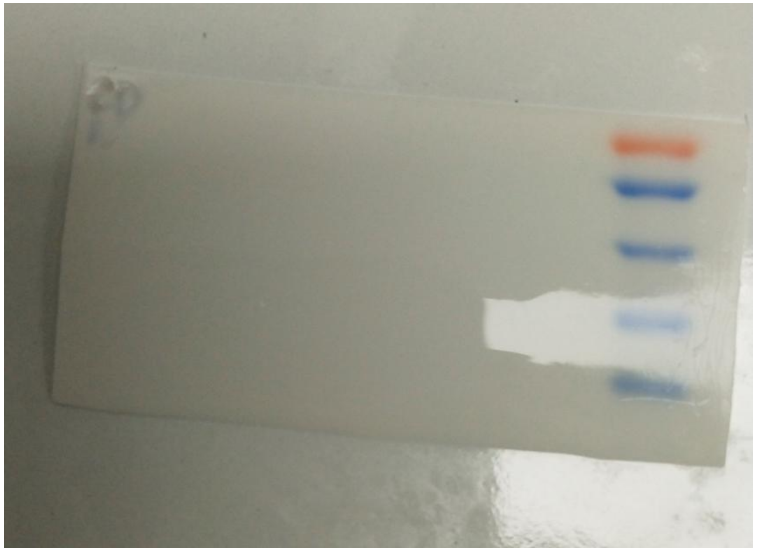

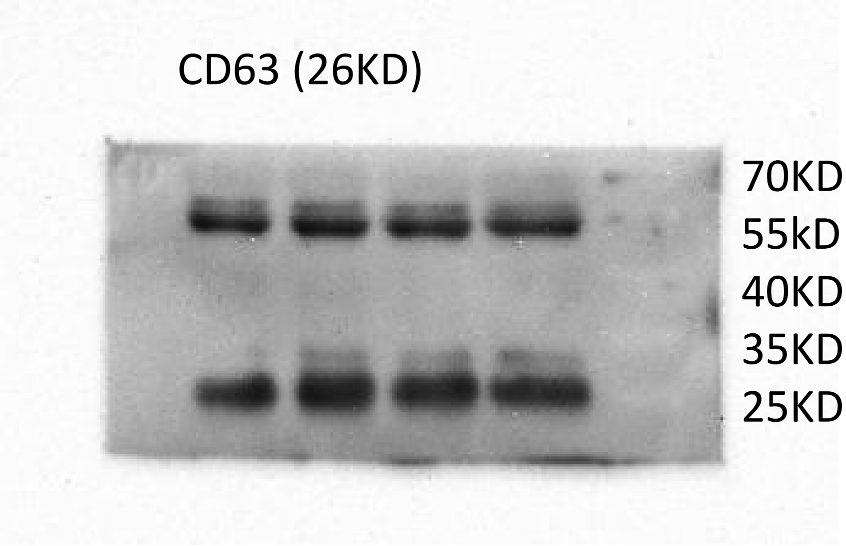
CD63 (26KD)
70KD
55kD
40KD
35KD
25KD

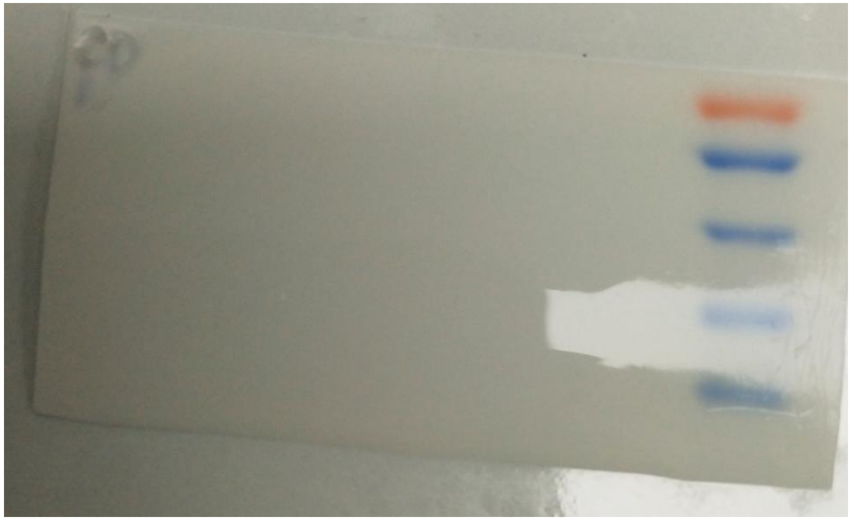

TSG101 (44KD)

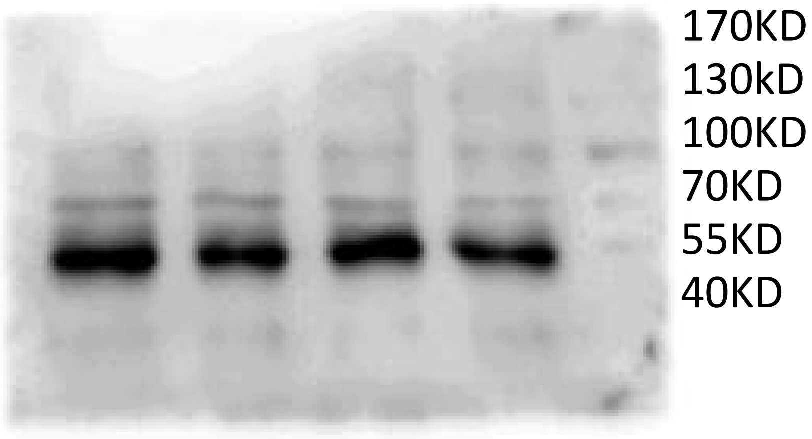

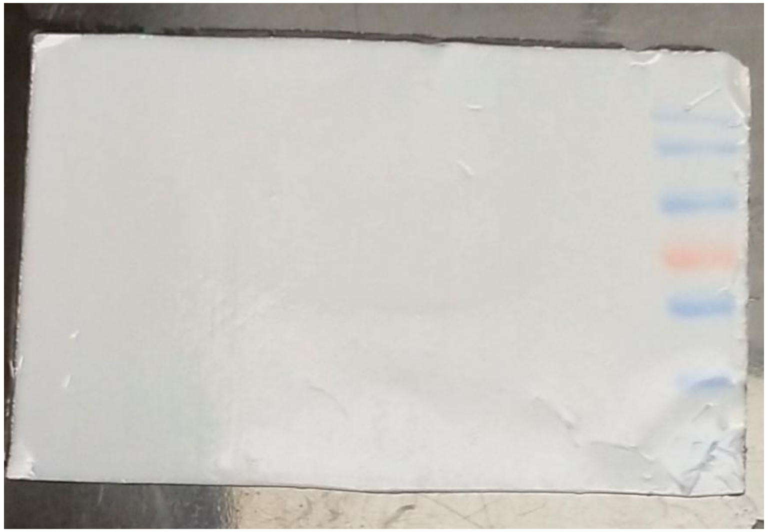

CBL (120KD)

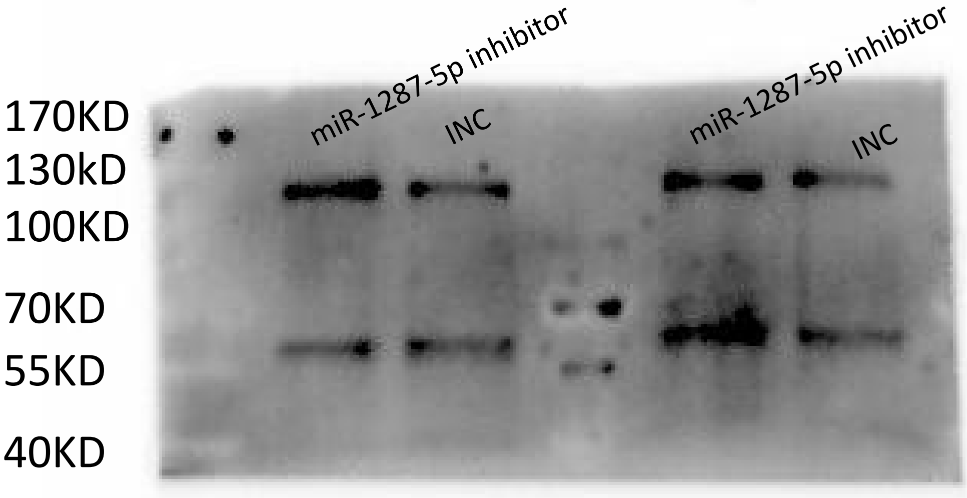

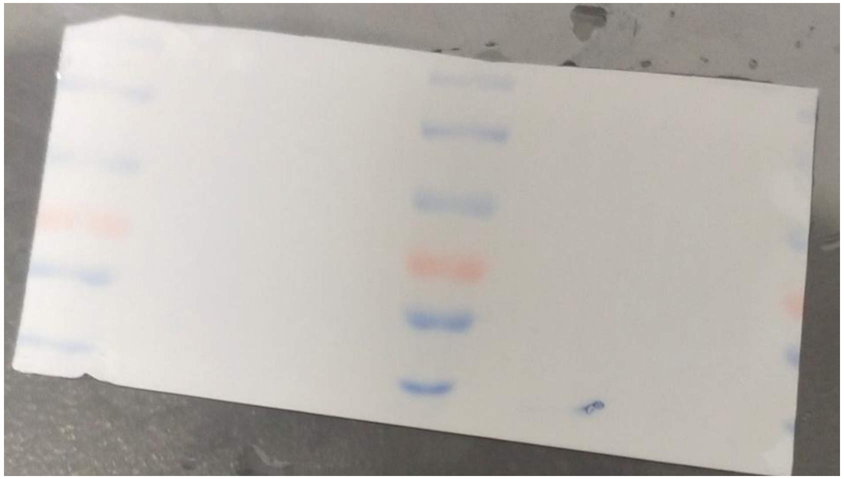

β-tublin (55KD)

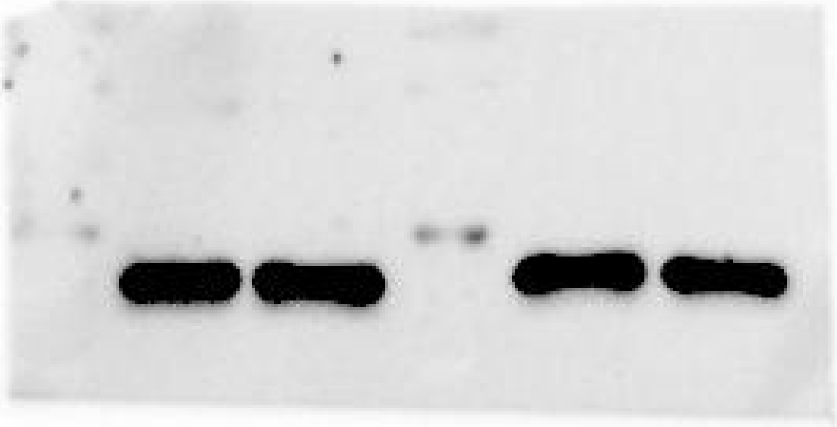

CBL (120KD)

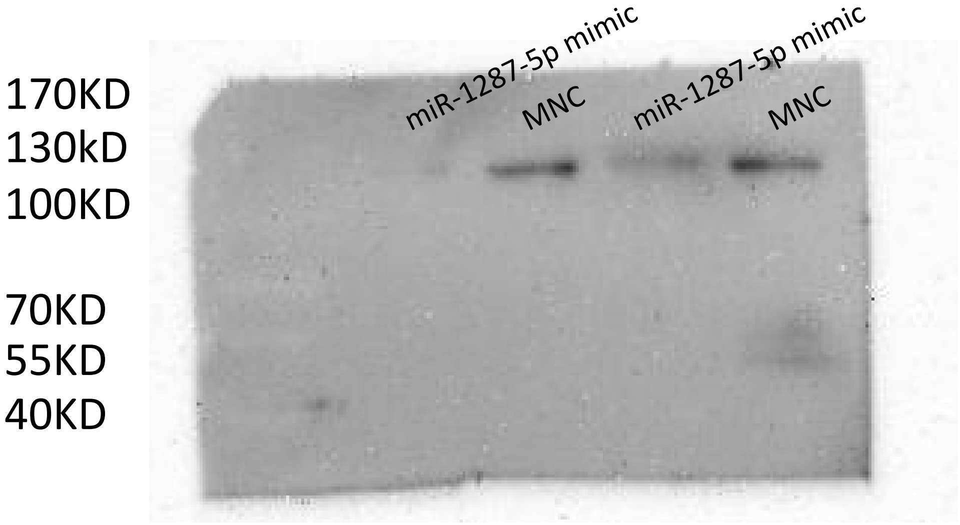

β-tublin (55KD)

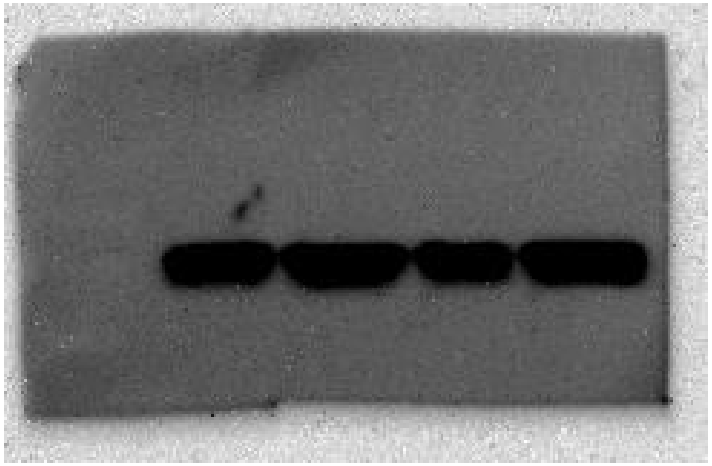

CBL (120KD)

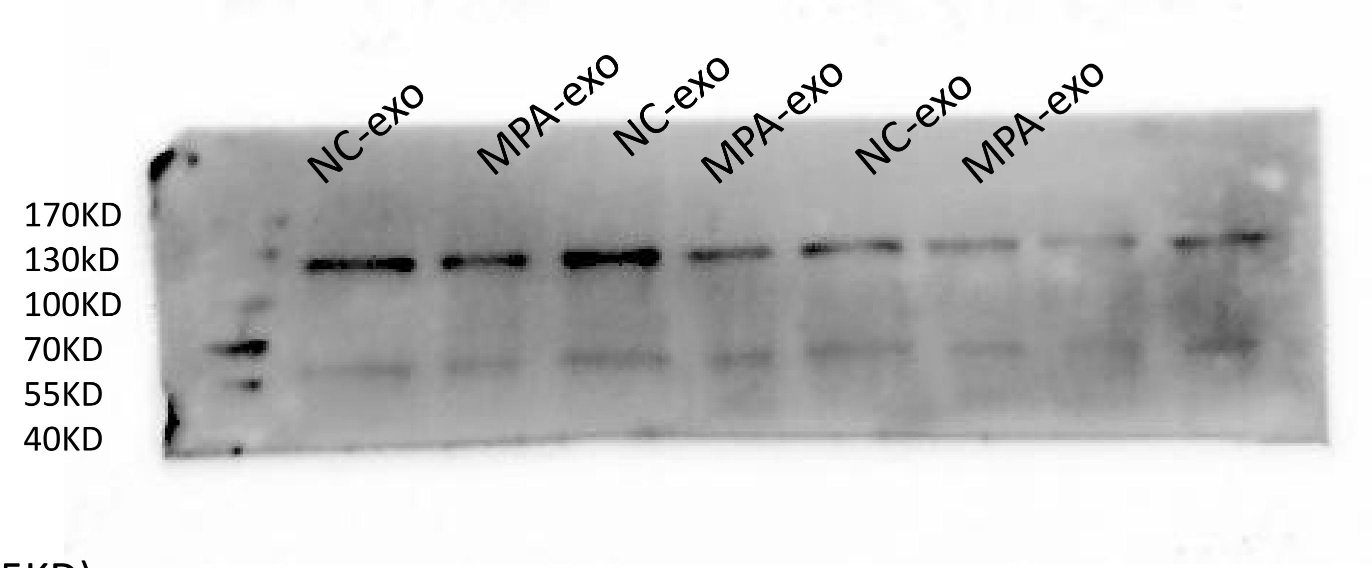

β-tublin (55KD)

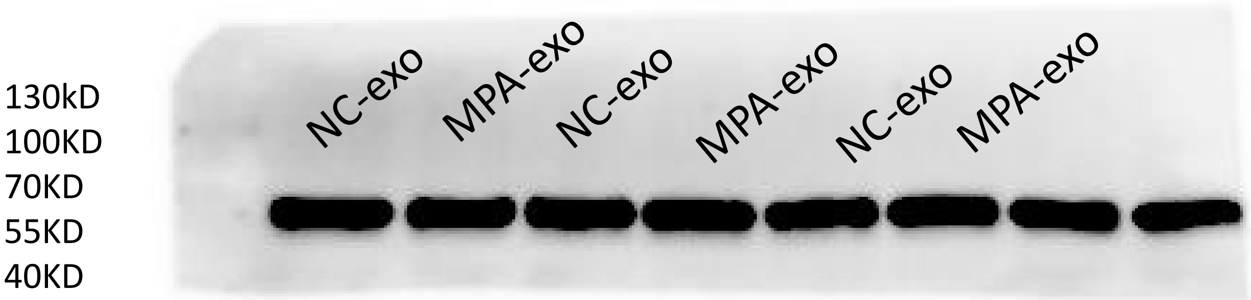

Supplement: Supplemental Information 2 [file peerj-11-14579-s002.pdf]
